# The epidemiological trend of monkeypox and monkeypox-varicella zoster viruses co-infection in North-Eastern Nigeria

**DOI:** 10.3389/fpubh.2022.1066589

**Published:** 2022-12-15

**Authors:** Roland Stephen, Faith Alele, Jamiu Olumoh, Jennifer Tyndall, Malachy Ifeanyi Okeke, Oyelola Adegboye

**Affiliations:** ^1^Department of Internal Medicine, Modibbo Adama University Teaching Hospital, Yola, Adamawa, Nigeria; ^2^Public Health and Tropical Medicine, College of Public Health, Medical and Veterinary Sciences, James Cook University, Townsville, QLD, Australia; ^3^Australian Institute of Tropical Health and Medicine, James Cook University, Townsville, QLD, Australia; ^4^Department of Mathematics and Statistics, American University of Nigeria, Yola, Adamawa, Nigeria; ^5^Department of Natural and Environmental Sciences, American University of Nigeria, Yola, Adamawa, Nigeria; ^6^World Health Organization Collaborating Center for Vector-Borne and Neglected Tropical Diseases, College of Public Health, Medical and Veterinary Sciences, James Cook University, Townsville, QLD, Australia

**Keywords:** monkeypox virus, MPX, varicella zoster virus, Nigeria, coinfection

## Abstract

**Background:**

Monkeypox (MPX) is endemic in Nigeria, but it was first reported in Adamawa state, North-Eastern Nigeria, in January 2022. There are currently 172 cases of MPX in Nigeria, with four reported deaths, and Adamawa has the second-highest case count. Therefore, this study was undertaken to evaluate the epidemiological profile of this viral disease.

**Methods:**

This is a cross-sectional study. The skin and blood samples were screened for the presence for Monkeypox virus (MPXV) and Varicella Zoster virus (VZV) DNA by real-time PCR; the clinical diagnosis was based on symptoms of visual signs of skin lesions and other clinical symptoms from January to July 2022.

**Results:**

A total of 33 suspected cases aged 1–57 years [26 (79%) males vs. 7 (21%) females] were screened for MPX and VZV. Twenty-four (72.7%) were positive (6.1% were MPX only, 39% were VZV only, and 27% were both MPX and VZV). Most cases of MPX (82%), VZV (69%) and MPX-VZV co-infection (78%) occurred in males. More than half (54%) of those infected were children and adolescents between 0 and 19 years. All patients experienced body rashes and itching, and other clinical symptoms included fever, headache, mouth sores, muscle aches and lymphadenopathy. Over 64 and 86% of patients had contact with livestock and rodents, respectively.

**Conclusion:**

MPXV, VZV and MPXV-VZV co-infections occurred predominantly among males and children in Adamawa state, Nigeria. Given the patient contact with rodents and livestock, further research on the animal reservoir is needed to highlight the transmission of MPXV in Adamawa.

## Introduction

Human monkeypox (MPX) is a viral zoonosis with symptoms similar to those seen in smallpox, except for lymphadenopathy (1). It is also clinically less severe than smallpox (2), but the disease may be fatal in individuals with compromised immunity. Monkeypox virus (MPXV), a double-stranded DNA virus that belongs to the genus *Orthopoxvirus*, is endemic in the Congo basin and West African countries (1, 3). Studies have shown that in areas where MPXV and varicella zoster virus (VZV) are circulating, VZ is often misdiagnosed as MPX and vice versa (4, 5). VZV is the virus that causes chickenpox and is an alpha herpes virus of the same subgroup as herpes simplex virus (HSV) 1 and 2 (6).

The current global spreading of cases of monkeypox to non-endemic countries has been linked to the 2017–2018 outbreaks in Nigeria (7). The outbreak began in 2017 (8, 9) and was the largest monkeypox outbreak in Nigeria's history, with a total of 189 confirmed MPX cases from September 2017 through November 2019 across 18 states (8–10). As of 4^th^ November 2022, 78,229 confirmed cases of MPX have been recorded in 109 countries globally, with 77,301 (98.8%) in locations that have not previously reported MPX (11). A total of 41 deaths have been recorded thus far (case fatality rate of 0.05%). On 23^rd^ July 2022, the world health organization (WHO) declared the MPX outbreak a Public Health Emergency of International Concern (12, 13). Since the beginning of the current outbreak, the Americas and Europe regions have been the worst hit, accounting for 98.4% of the global cases (The United States of America alone, with 28,657 cases, accounting for more than one-third of the global burden of the disease). Although Africa has only recorded 937 (1.2%) cases of MPXV, it accounted for 34.1% of the worldwide mortality from the virus (11).

From 1^st^ January to 14^th^ August 2022, Nigeria has reported 220 confirmed cases of MPX and four deaths (case fatality rate of 1.8%), of which Adamawa state accounts for 13 cases and zero death (14). Although MPX is endemic in Nigeria, there was no report of the incidence of the disease in the North-Eastern state of Adamawa until January 2022. Pembi et al. (15) reported the first confirmed case of MPX in Adamawa. However, the study did not evaluate the epidemiological profiles of MPX cases in the state nor document the evolving epidemiological dynamics of MPX, particularly with regard to gender susceptibility, age range, MPX-VZV co-infection and human behaviors that may influence zoonotic spillover, and human-human transmission events. In some cases, clinical analysis of suspected human MPX is associated with cases of co-infection with MPXV and VZV, but there appear to be no epidemiologic linkages in the transmissions (16). Although MPX and Chickenpox have been diagnosed in the same individuals in other countries (4, 16), there seems to be no report of such co-infections in Nigeria, particularly in Adamawa state. Thus, this study aims to provide details on epidemiological and clinical characteristics of cases of MPX, VZ and co-infections with their respective etiological agents.

## Materials and methods

### Study area, design, and data collection

**Figure 1:**
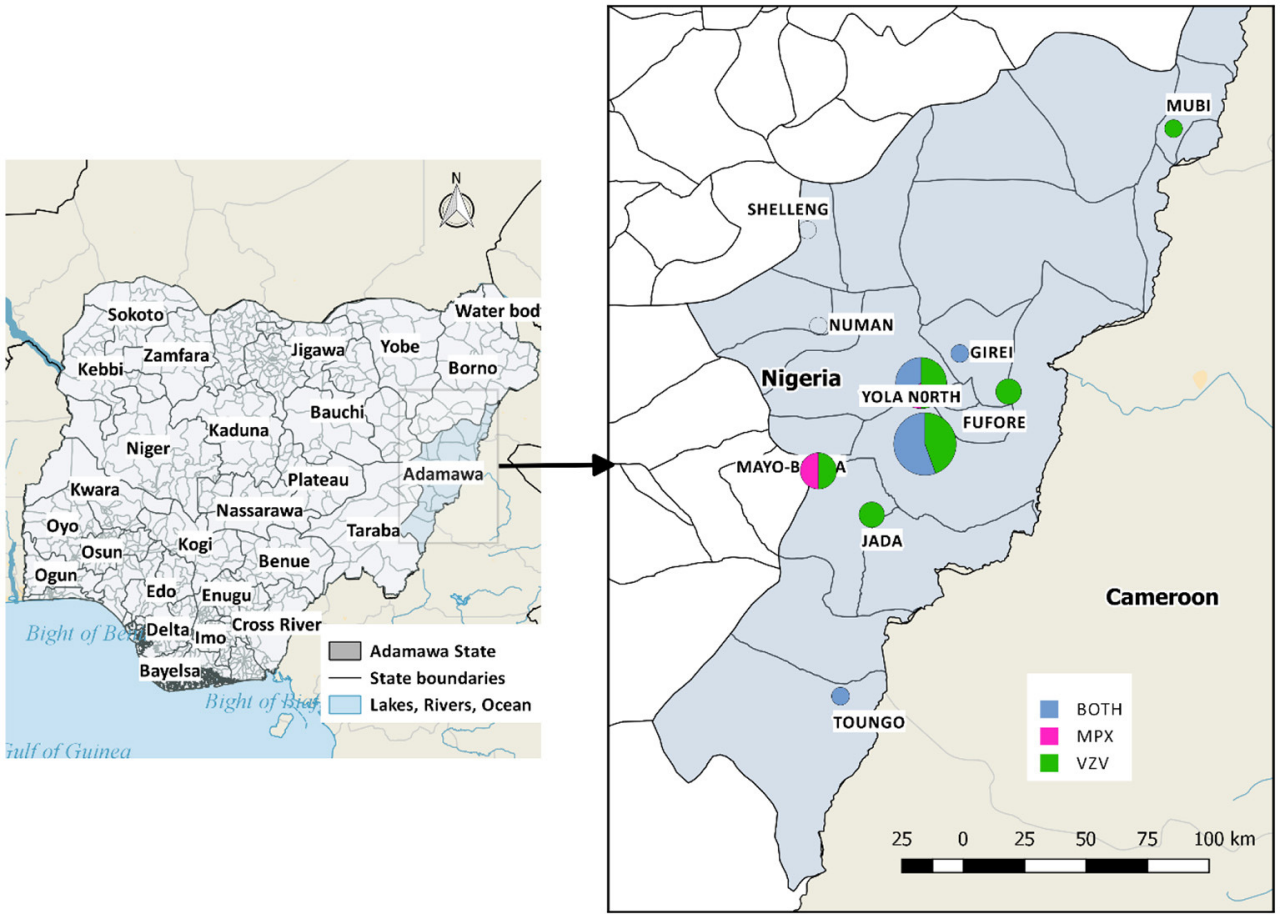
Map of the study area, with the affected local government areas of Adamawa State. Positive cases of MPX and/or VZ were reported in Mubi (1), Girei (1), Yola North (8), Yola South (12), Fufore (2), Mayo-Belwa (4), Jada (2), Toungo (1). Shelleng and Numan have suspected cases but tested negative.

This study utilizes data from suspected and laboratory-confirmed cases of MPX and VZV in Adamawa State, North-Eastern Nigeria. Adamawa is one of Nigeria's largest states, occupying about 36,917 square kilometers and borders Borno state to the North-West, Gombe state to the West, Taraba to the South-East, and Cameroon to the East (Figure 1). The study sites are located between latitudes 9.28–9.30°N and longitudes 12.454–12.458°E in this state.

### Data and surveillance system

This study was cross-sectional. Data were collected using two approaches. Firstly, routinely collected secondary surveillance data was utilized to estimate the prevalence of MPX, VZ and co-infections. Secondly, participants were approached to collect information on detailed exposure and outcome.

#### Surveillance system

Following grassroots community sensitization on the outbreak and public education on the symptoms of MPX by the disease surveillance and notification officers (DSNOs) and electronic media, the sensitized locals reported suspicious cases to DNSOs or the nearest primary health care center. The trained DSNOs, trained on sample collection, collected blood sample specimens and vesicular/ pustule swabs, filled out the case investigation form (CIF), assigned a unique number to the patient and also labeled the specimen. The state epidemiologist and the Nigerian Center for Disease and Control (NCDC) were notified simultaneously. The samples were collated at the state focal laboratory, processed and couriered to the NCDC National Reference Laboratory, Abuja, for laboratory confirmation. Laboratory results are communicated to the state epidemiologist and the state focal laboratory scientist *via* email within 48–72 h. From thence, the respective DSNO and the patient are subsequently informed. Meanwhile, the patient is referred for care at the nearest public healthcare facility while the patient's close contacts are placed under watch (17).

#### Case definition

##### Suspected cases

A person is suspected of having MPX if the following symptoms are noted. An acute illness with fever >38.3^o^C, intense headache, lymphadenopathy, back pain, myalgia, and intense asthenia followed 1–3 days later by a progressively developing rash often beginning on the face (most dense) and then spreading elsewhere on the body, including soles of feet and palms of the hand (1).

##### Probable case

A case that meets the clinical case definition but is not laboratory-confirmed and has an epidemiological link to a confirmed case.

##### Laboratory investigation of confirmed cases

Suspected cases of MPX were reported to the DNSO by local community or during surveillance in which the DNSO came across individuals manifesting pox-like skin rashes, fever and other probable symptoms. The DSNO immediately activates the standard protocol for case investigation as laid down by NCDC (17). A clinically compatible case of MPXV is one that is laboratory confirmed by RayBiotech MPXV real-time Polymerase Chain Reaction (PCR) protocol, while that of VZV was tested using a real-time in-house PCR assay at the National Reference Laboratory (17).

#### Cross-sectional survey

To better understand the patient dynamics and possible exposure site, a follow-up survey was created to investigate details of recovery, care and symptoms among patients. The survey consisted of questions on socio-demographics, symptoms, exposure, presence of comorbidities, and sexually transmitted infection, respectively. The survey was created in English, and enumerators (first author and local health workers) interviewed the patients in the local language when necessary. Data collection took place from 1^st^ to 11^th^ September 2022.

### Data analysis

Deidentified data were analyzed and visualized in R version 4.0.1. Patient demographics and clinical presentation (symptom and exposure) were aggregated and presented as frequencies and percentages for categorical variables and median and interquartile ranges for continuous variables.

### Ethics approval and consent

The National Health Research Ethics Committee approved the study with protocol number NHREC/01/01/2007-25/08/2022 and approval number NHREC/01/01/2007-31/08/2022. All respondents gave verbal consent. In addition, written informed, and signed consent was obtained from study participants for the collection of images.

## Results

**Figure 2:**
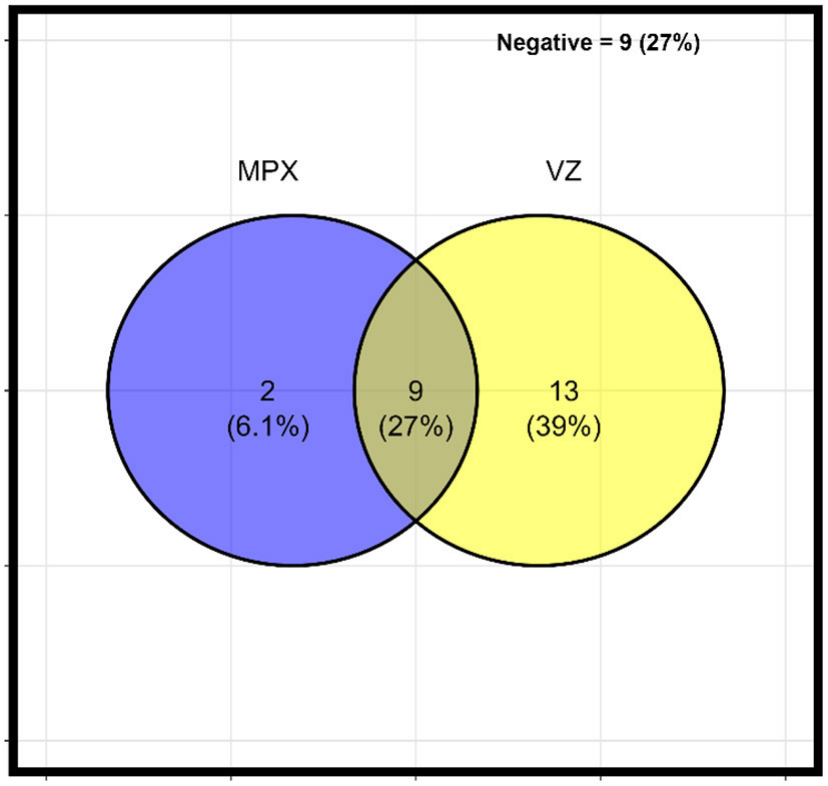
Distribution of clinical diagnosis of MPX and/or VZ in Adamawa state.

Detailed study demographic characteristics are listed in Table 1. Males accounted for 79% (26) of the total population, 54% were children and adolescents between the ages of 1 and 19 years (24% between 1 and 9 years, and 30% between 10 and 19 years). A high proportion of the suspected cases investigated were either confirmed (MPXV, VZV or both MPXV and VZV). Of the 33 suspected cases, 27% (9) were positive for both MPXV and VZV, 6.1% (2) were MPXV only, 39% (13) were VZV only, while only 9 (27%) cases were negative or inconclusive (Table 1 and Figure 2). More than 1-in-2 of the VZV patients had a presence of crusting around lesions compared to 1-in-10 observed among MPXV and VZV. The average days from onset of fever to onset of rash was 2.9 days (SD = 4.1), which was longer among VZV only, 5.5 days (5.7) than MPXV and VZV co-infected patients, 1.2 days (1.2).

**Table 1:** Patient demographic and clinical characteristics in all case series.

**Characteristics**	**Total**	**All MPX**	**MPX only**	**All VZ**	**VZ only**	**Co-infection**	**Negative/inconclusive**
*n* (*N*)	33	11/33	2/33 (6.1%)	22/33 (67%)	13/33 (39%)	9/33 (27%)	9/33 (27%)
Age, median (IQR)	14 (8–30)	14 (10–28)	14 (8–20)	13 (10–28)	12 (7–22)	14 (11–29)	30 (17–36)
**Age group**, ***n*** **(%)**							
0–9	8 (24)	2 (18)	1 (50)	5 (23)	4 (31)	1 (11)	2 (22.2)
10–19	10 (30)	4 (36)	0 (0)	9 (41)	5 (38)	4 (44)	1 (11.1)
20–29	4 (12)	3 (27)	1 (50)	3 (14)	1 (7.7)	2 (22)	0 (0)
30–39	7 (21)	2 (18)	0 (0)	3 (14)	1 (7.7)	2 (22)	4 (44.4)
40+	2 (6.1)	0 (0)	0 (0)	2 (9.1)	2 (15)	0 (0)	0 (0)
Missing	2 (6.1)						2 (22.2)
**Sex**, ***n*** **(%)**							
Male	26 (79)	9 (82)	2 (100)	16 (73)	9 (69)	7 (78)	8 (88.9)
Female	7 (21)	2 (18)	0 (0)	6 (27)	4 (31)	2 (22)	1 (11.1)
Presence crust, *n* (%)	8 (24.2)	NA	NA	NA	7 (53.9)	1 (11.1)	NA
Days,^†^ [mean (SD)]	2.9 (4.1)	NA	NA	NA	5.5 (5.7)	1.2 (1.2)	NA

† Average days between the onset of fever and rashes.

NA, not available.

**Figure 3:**
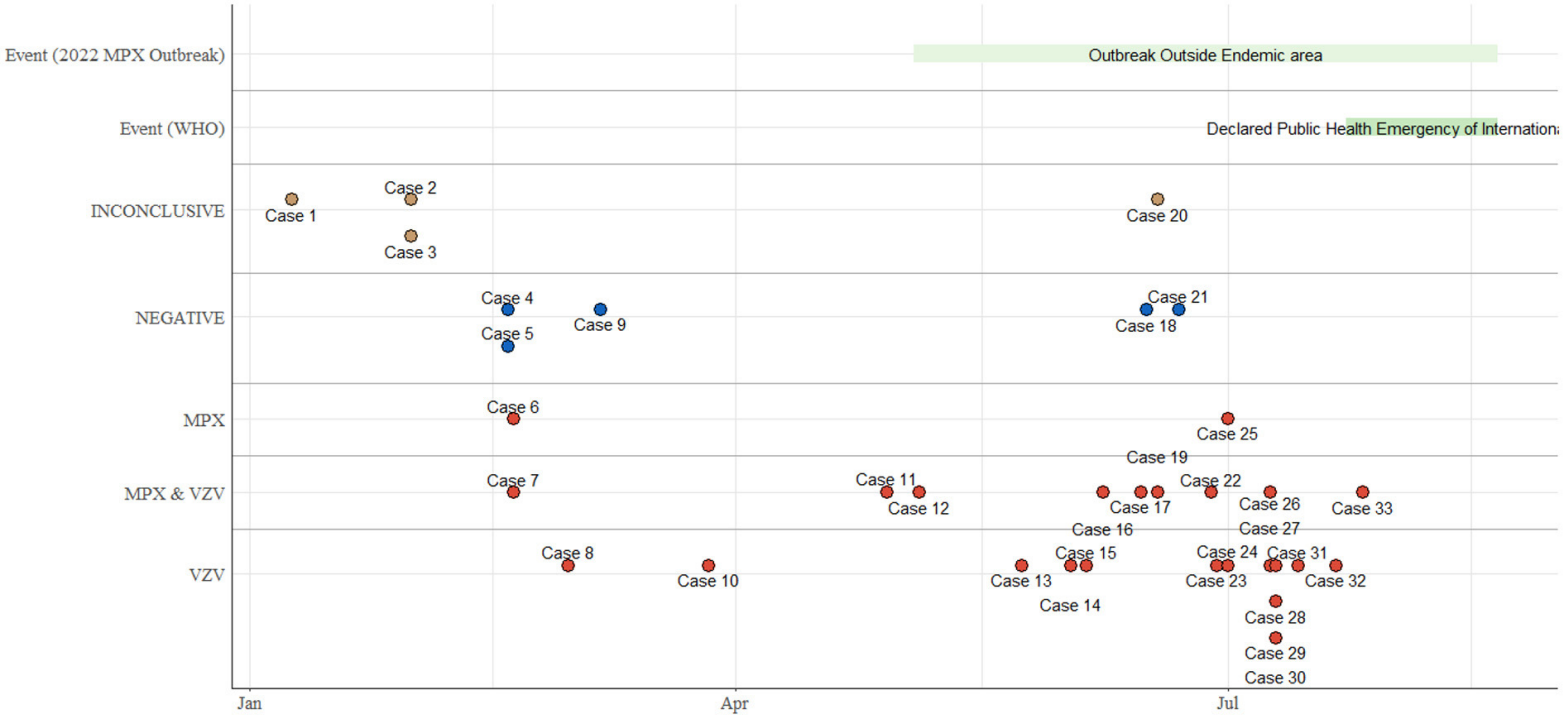
Epidemiological timeline of the outbreak of MPX in Adamawa State.

The first suspected MPX case in Adamawa was reported on 9^th^ January 2022, followed by the second and third cases on 31^st^ January 2022. The results of these three cases were inconclusive. Case 6 was the first confirmed case whose rashes began on 19^th^ February 2022. Thereafter, there was an increase in MPX and/or VZ cases from May through July, coinciding with the 2022 outbreak outside the endemic region (Figure 3).

Furthermore, there are three clusters of MPX observed in this study, two household clusters and one correctional facility (Prison) cluster. The two household clusters comprise children aged 2–14 years who were suspected of MPX after presenting symptoms such as fever and rashes. Case 7, the youngest of three siblings (Cluster 1), a household exposure with Case 6 and 33, tested negative for both MPXV and VZV, while case 33 was infected with both MPXV and VZV, and case 6 tested positive for MPXV only. However, all siblings (cases 14, 15, and 17) in the second cluster 2 tested positive for VZV only. The third cluster were male inmates of a correctional facility aged (22–30 years). Among the four inmates in this cluster (Cases 22, 24, 25, and 27), one tested negative for both viruses, one positive for MPXV, one was co-infected with both viruses and the last tested positive for VZV.

**Figure 4:**
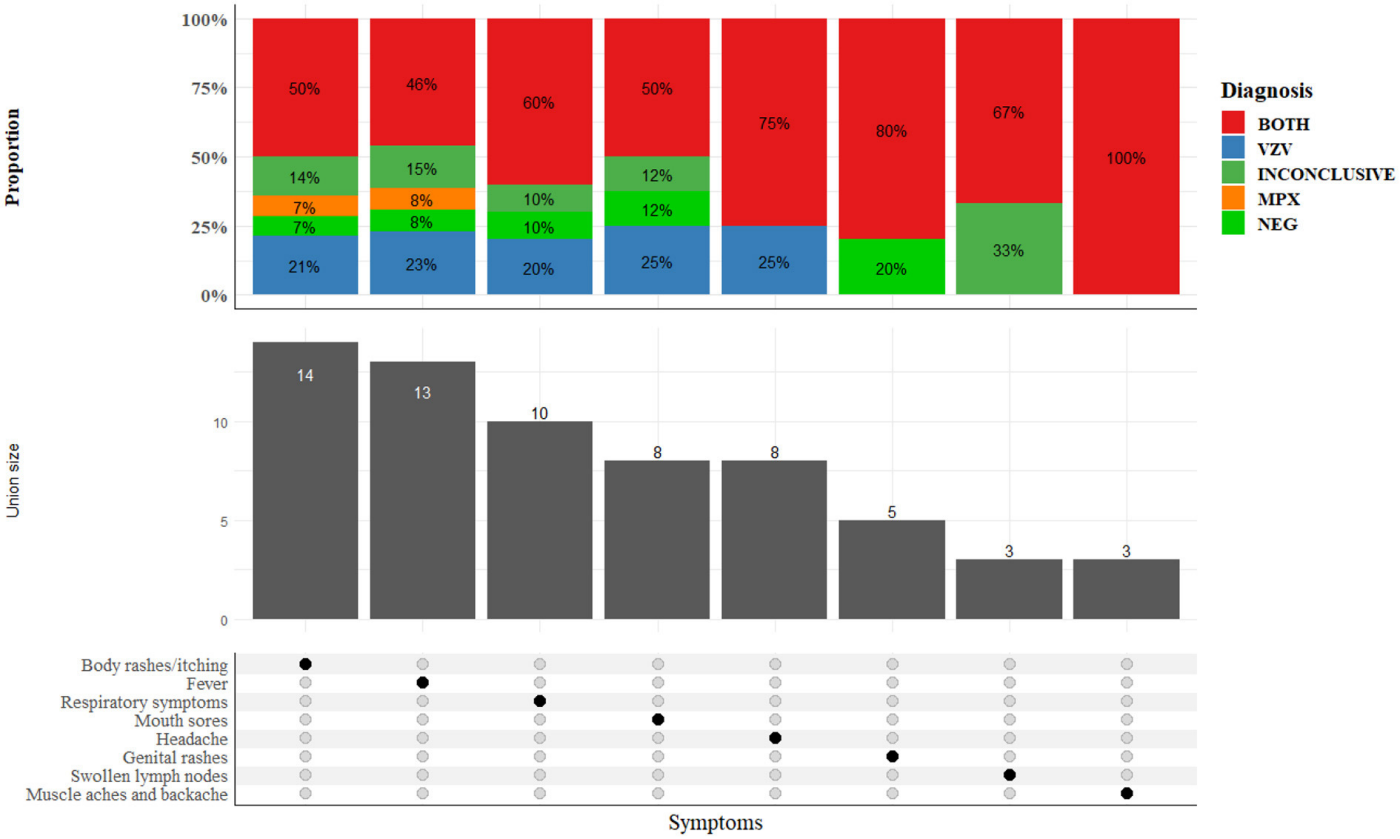
Clinical characteristics among 14 patients included in the follow-up survey.

**Figure 5:**
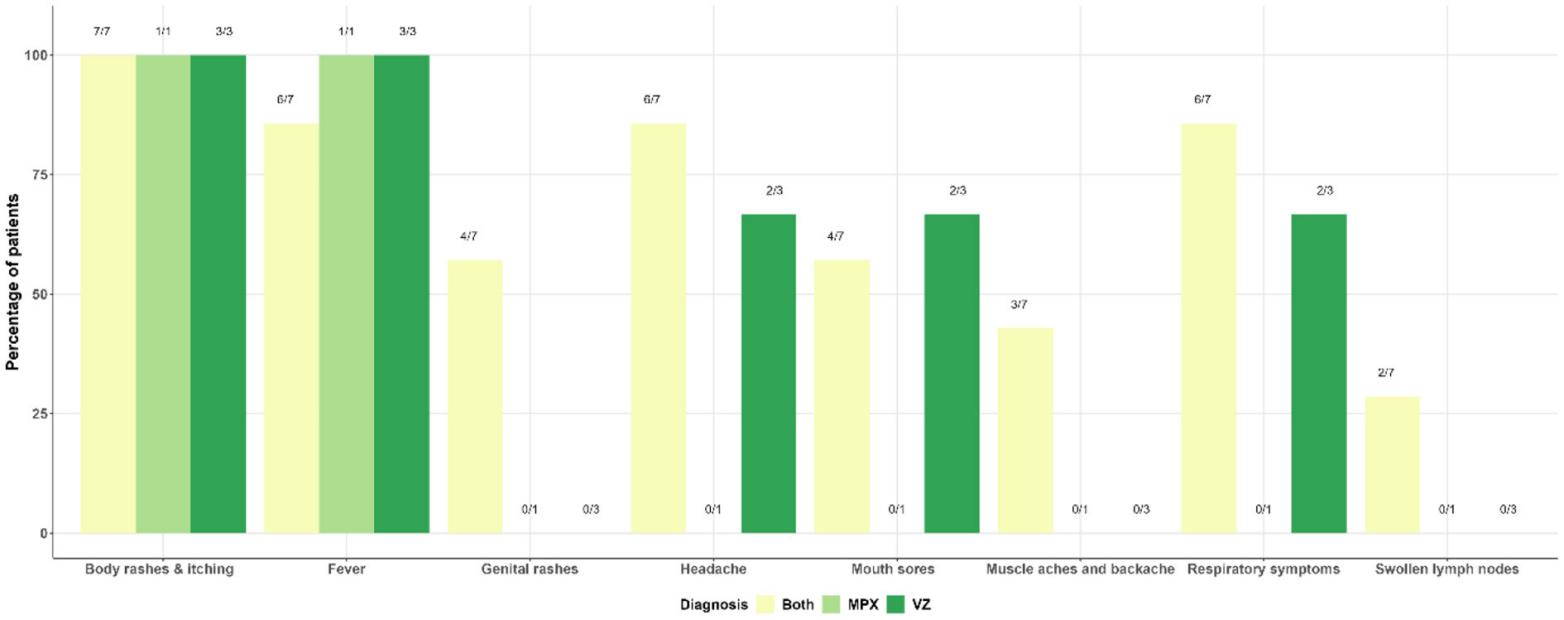
Distribution of symptoms among patients diagnosed with MPX, VZ, and both.

The distribution of characteristics of cases in the follow-up survey is presented in Figure 4. Although all 14 patients surveyed had body rashes (skin lesions), half were confirmed as both MPX and VZ cases, 3 (21%) had VZ only, 1 MPX only, 2 (14%) were inconclusive, and one tested negative for both MPXV and VZV. Other major clinical features reported by the patients include fever (13/14) and respiratory symptoms such as cough (10/14). Genital lesions were also reported in 5 out of the 14 cases interviewed. Supplementary Figure 1 presents a combination of clinical characteristics among patients for up to three symptoms at a time. The distribution of symptoms among patients diagnosed with MPX, VZ and con-infection is presented in Figure 5. Notably, body rashes and itching are equally common among patients diagnosed with MPX, VZ or both. Although only two patients were diagnosed with MPX only, in addition to body rashes and itching, they only displayed fever. Two-thirds of VZ patients had headaches, mouth sores and respiratory symptoms. Only co-infected patients (3 out 7) reported muscle aches and backaches.

**Figure 6:**
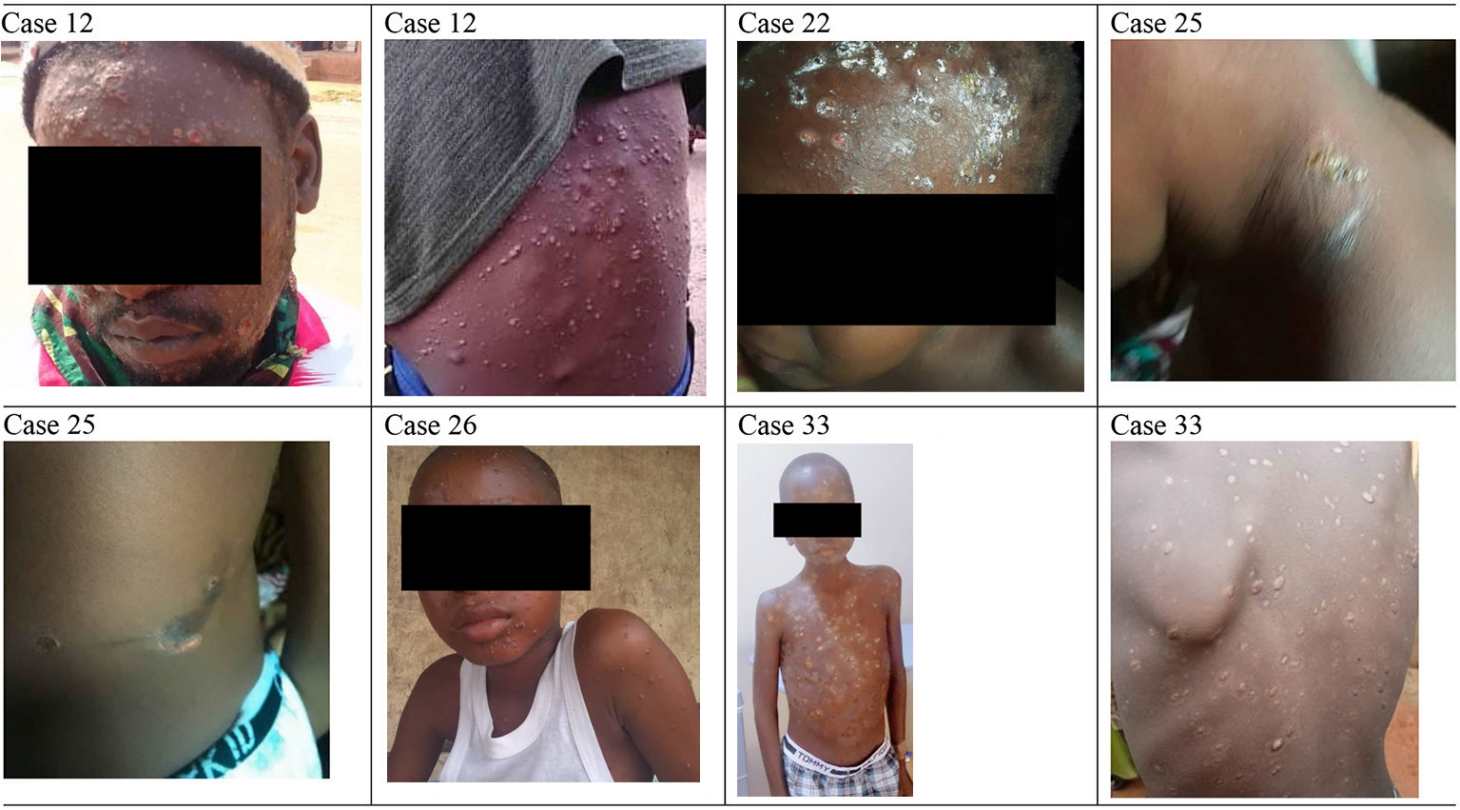
Spectrum of skin lesion observed in follow-up patients. Case 12 (MPX and VZ), Case 22 (MPX and VZ), Case 25 (MPX), Case 26 (MPX and VZ), and Case 33 (MPX and VZ).

Figure 6 presents the spectrum of skin lesions observed in selected patients ranging from vesicular and pustular to crusted lesions, while symptoms distribution across age groups are presented in Supplementary Figure 2. All 14 patients surveyed reported body rashes and itching when comparing symptoms across age groups. However, fewer children aged 0–9 reported genital rashes, headaches, mouth sores, muscle aches and backaches, respiratory symptoms, and swollen lymph nodes.

**Figure 7:**
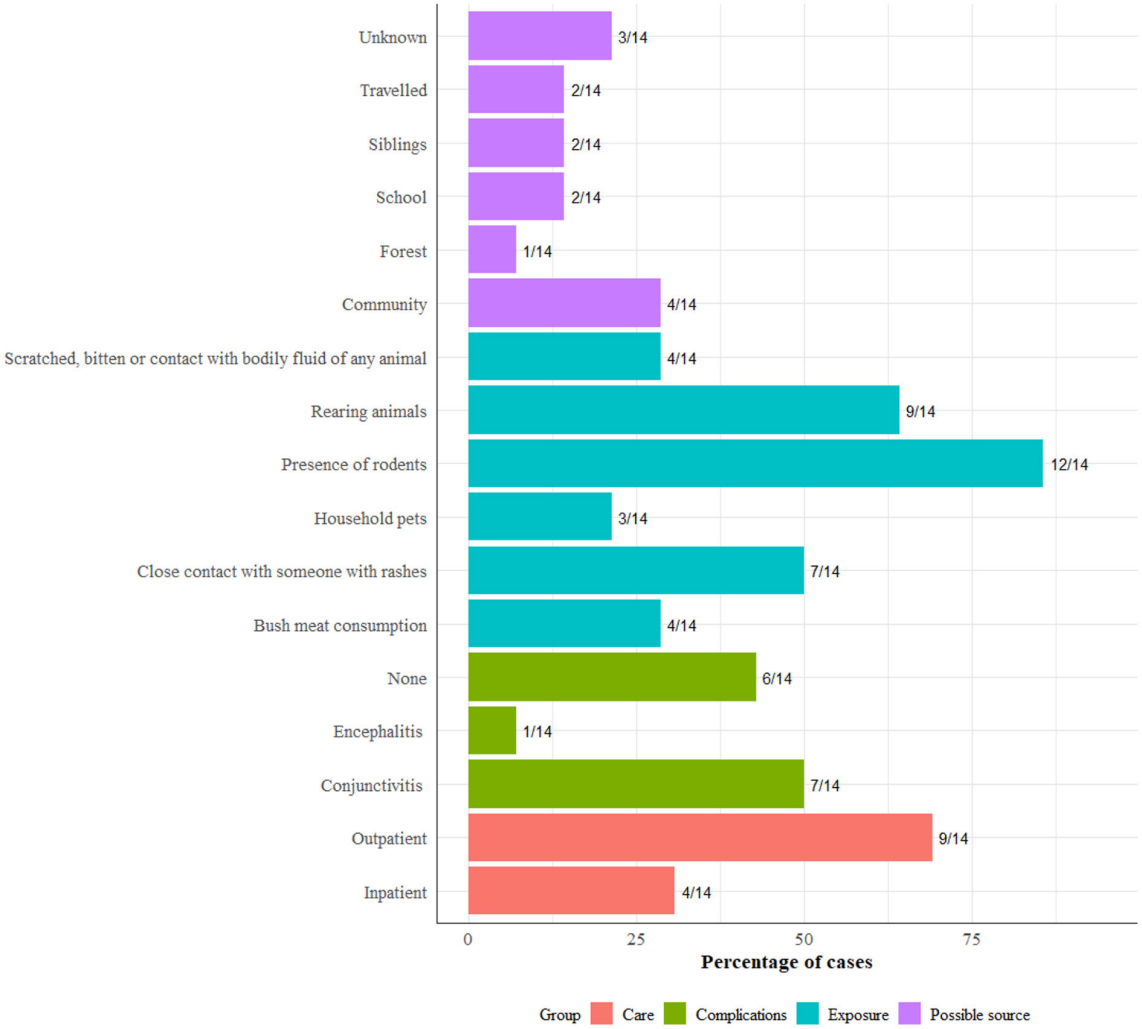
Exposure, complication, care type, and comorbidities among 14 patients included in the follow-up survey.

Exposure and possible sources of infection included community exposure in 4 (28%), household exposure in 2 (14%), school exposure in 2 (14%) and travel (14%) (Figure 7). About 86% (12/14) of the patients surveyed reported the presence of rodents in and around households, while half reported contact with someone with rashes. A high number of patients, 9/14 were treated as outpatients, and half, 7/14 showed symptoms of conjunctivitis.

All patients were managed as outpatients and treated symptomatically with antibiotics, pain relievers, antipruritic medications and multivitamins. Approximately 20% of them were admitted, isolated and managed on similar medications but with the addition of intravenous fluids (IVF) and with strict adoption of barrier nursing by trained healthcare workers. The average hospital stay was about 10 days. After discharge, they subsequently had follow-up visits as required.

## Discussion

This study evaluated the prevalence of MPX, VZ and MPXV-VZV co-infections and the epidemiological characteristics of the co-infection in Adamawa State, Nigeria. Findings showed a lower prevalence of MPX only, a high prevalence of MPX and VZ concurrent diseases and a high prevalence of VZV infection. A large proportion of the participants reported the presence of rodents in and around the household.

While MPX is endemic to Nigeria, other diseases that present with rashes, such as cutaneous anthrax, fungal infections in HIV patients, or VZ, may be inadvertently captured as MPX during surveillance (16, 18). The findings of this study showed a high prevalence of MPXV and VZV co-infection and VZV infection in the study population. Previous studies from the Democratic Republic of Congo have also reported cases of MPXV and VZV co-infection (4, 16). While the biological plausibility for the coexistence of both viruses is unclear, one explanation is that latent VZV infection is activated by an acute infection of MPXV (16). On the other hand, it is also possible that the lesions associated with VZV infection may have been present before MPX and served as a portal of entry for MPXV (4). However, it was difficult to determine which occurred first in our cohort of participants. Possibly, there may have been some exposure to the disease (MPX) *via* zoonotic transmission (19). Some participants reported being in contact with animals before the onset of the illness. Nonetheless, this requires further exploration.

In the 2017–2020 outbreak, the MPX disease predominantly affected the adult age group 21–40 years, who are naturally more exposed to MPXV reservoir hosts such as wild rodents (9). The disease burden was predominantly among males and children in this study. The skewness of MPX toward the male gender may be because most household settings in Nigeria are highly patriarchal (20), where males are generally the breadwinners (20) and risk-takers, thus, at higher risk of exposure to infected animal and human hosts (21). While the current outbreak suggests increased infection among persons who are gay or bisexual, the population sampled in this study all identified as heterosexual (22). On the other hand, the predominance of MPX among children in this study may largely be due lack of preexisting immunity derived from active (vaccination) and natural exposure to orthopoxviruses. Besides being physiologically of suboptimal immunity, this cohort did not have the smallpox vaccination, which confers about 85% cross-immunity against MPXV.

Interestingly children and adolescents had the highest proportion of the co-infection in our study. This finding is in consonance with previous studies where children were reported to have a higher prevalence of co-infection (4, 16). However, the idea that VZV may have been reactivated in children seems less likely. Primary infection with VZV resulting in chickenpox is more often seen in children 1–9 years than adults (23). Thus, it is possible that the presence of VZV in children and adolescents in the study could have been a primary infection. It is also possible that infection by either MPXV or VZV weakens the child/adolescent immune system more than in adults, making it likely for superinfection with either of the two viruses to occur more in children than in adults. More research is required to determine and distinguish between the onset of the diseases as well as determine viral and host factors that modulate MPXV-VZV co-infection.

While co-infection of MPXV and VZV was reported, infection with VZV only was found in ~40% of the study population. The infection was more prevalent in children and adolescents, which buttresses current evidence. VZV is a highly contagious virus, and is a member of the herpesvirus family, with humans as the only reservoir (24). It can spread from person to person *via* direct contact with respiratory droplets or aerosols (24). Considering its virulence (24), it is not surprising to see its high prevalence in children in this study. Children typically play together, share toys and touch each other during play, making it easy to spread infections (25). In addition, some children in the study are siblings, highlighting the spread *via* close family contact (24). Furthermore, the *Varicella* vaccine is not included as part of the routine vaccines on Nigeria's childhood immunization schedule (26). Thus, children who have not been vaccinated are at an increased risk of contracting the disease.

Given that the clinical characteristics of VZV are similar to MPX, hence the misdiagnosis at initial assessment in the study population. Documented evidence also shows that VZV infection is frequently misdiagnosed as MPXV infection in regions where both viruses are circulating (16, 27, 28). In addition, the observation that MPX cases were found in the age groups < 40 means that they have not been vaccinated against smallpox since the smallpox vaccination ceased in 1980. Consequently, the increasing incidence of MPX in Adamawa, Nigeria and globally may be partly due to a wane in population immunity to orthopoxviruses (OPXV) (29).

### Implications for practice and future research

The study demonstrated that both infections occur more in younger people. However, the biological mechanism that underpins the coexistence of both infections is poorly understood. Further research is required to comprehensively understand the epidemiology of MPXV-VZV co-infection to develop strategies to limit the distribution of the diseases among individuals and to find appropriate treatment strategies for co-infected patients. Because MPX can be a self-limiting and mild disease in some instances, some cases never get reported as they are mistaken for or misdiagnosed as chicken pox. This again reinforces the need to urgently establish an active surveillance system for MPX and VZV to pick transmissions occurring in communities.

In addition, it may be worthwhile for the chickenpox vaccine to be added to the routine vaccines for children. While the vaccine may be available in Nigeria, it is not listed as a routine immunization for children (26). Adding the chickenpox vaccine to the routine immunization list may reduce the incidence of VZV in children. Furthermore, there is limited genomic evidence on MPXV and VZV co-infection. Future studies could consider conducting comparative functional genomic analysis on MPXV and VZV co-infection and superinfection.

Deforestation, desert encroachment, rising poverty level and humanitarian crises arising from conflicts and natural disasters are implicated in the resurgence of several zoonotic diseases, such as MPXV and Ebola (10, 30, 31). Uncontrolled development, rural-urban drift, and urbanization disturb the natural ecosystem and displace animals from their natural habitat (32). Consequently, these displaced animals establish new ecological niches among humans, thereby setting the premise for spreading zoonotic diseases. Given the zoonotic nature of MPX, the relevant agency must be utilized in a One Health approach to combat the spread of the disease. Capacity building of existing institutions like the veterinary, game reserve and animal husbandry is required to establish a mechanism for strategic and extensive surveillance of MPXV.

### Limitations

It is important to highlight the limitations of this study. First, we could not establish causal relationships due to the study design. In addition, this study was based on the number of reported cases in the state. Therefore, more research is needed with a larger sample size. Second, there was also a potential for recall bias to have been introduced as participants were required to recall events related to the disease. Third, we could not reliably ascertain the origin of these infections in this study. The unusually high frequency of contact with rodents and livestock observed amongst the suspected group, and probable community human-to-human transmission suggests a sustained possibility of an increase in the rate of transmission. However, further research on the animal reservoir is needed to highlight the transmission of MPXV in Adamawa.

## Conclusion

In summary, the findings of this study showed that MPX and VZV co-infection is prevalent and requires further investigation. In addition, this study highlights the need for active MPX and VZ surveillance systems in the country to enhance the early detection and control of the two viruses. Furthermore, given that MPX is a zoonotic viral disease, further research on the animal reservoir is critical, while strategic actions and interventions to ensure that the disease is not established in domesticated livestock and small animals are introduced.

## Data availability statement

The original contributions presented in the study are included in the article/Supplementary material, further inquiries can be directed to the corresponding author.

## Ethics statement

Written informed consent was obtained from the individual(s), and minor(s)' legal guardian/next of kin, for the publication of any potentially identifiable images or data included in this article.

## Author contributions

RS: conceptualized. RS, FA, JO, and OA: designed this study. FA and OA: designed the methodology. RS, JO, and JT: designed and assembled the patient database. OA: analyzed data, interpreted, and presented the results. RS, FA, JO, JT, MO, and OA: wrote the manuscript. All authors have reviewed and approved this manuscript.
