# Corrigendum: The epidemiological trend of monkeypox and monkeypox-varicella zoster viruses co-infection in North-Eastern Nigeria

**DOI:** 10.3389/fpubh.2023.1256195

**Published:** 2023-07-24

**Authors:** Roland Stephen, Faith Alele, Jamiu Olumoh, Jennifer Tyndall, Malachy Ifeanyi Okeke, Oyelola Adegboye

**Affiliations:** ^1^Department of Internal Medicine, Modibbo Adama University Teaching Hospital, Yola, Adamawa, Nigeria; ^2^Public Health and Tropical Medicine, College of Public Health, Medical and Veterinary Sciences, James Cook University, Townsville, QLD, Australia; ^3^Australian Institute of Tropical Health and Medicine, James Cook University, Townsville, QLD, Australia; ^4^Department of Mathematics and Statistics, American University of Nigeria, Yola, Adamawa, Nigeria; ^5^Department of Natural and Environmental Sciences, American University of Nigeria, Yola, Adamawa, Nigeria; ^6^World Health Organization Collaborating Center for Vector-Borne and Neglected Tropical Diseases, College of Public Health, Medical and Veterinary Sciences, James Cook University, Townsville, QLD, Australia

**Keywords:** monkeypox virus, MPX, varicella zoster virus, Nigeria, coinfection

In the published article, there was an error in Table 1 as published. There were errors regarding the number of patients with negative/inconclusive results by age group and gender. The corrected Table 1 and its caption appear below.

**Table 1:** Patient demographic and clinical characteristics in all case series.

**Characteristics**	**Total**	**All MPX**	**MPX only**	**All VZ**	**VZ only**	**Co-infection**	**Negative/inconclusive**
*n* (*N*)	33	11/33	2/33 (6.1%)	22/33 (67%)	13/33 (39%)	9/33 (27%)	9/33 (27%)
Age, median (IQR)	14 (8–30)	14 (10–28)	14 (8–20)	13 (10–28)	12 (7–22)	14 (11–29)	30 (17–36)
**Age group**, ***n*** **(%)**							
0–9	8 (24)	2 (18)	1 (50)	5 (23)	4 (31)	1 (11)	2 (22.2)
10–19	10 (30)	4 (36)	0 (0)	9 (41)	5 (38)	4 (44)	1 (11.1)
20–29	4 (12)	3 (27)	1 (50)	3 (14)	1 (7.7)	2 (22)	0 (0)
30–39	7 (21)	2 (18)	0 (0)	3 (14)	1 (7.7)	2 (22)	4 (44.4)
40+	2 (6.1)	0 (0)	0 (0)	2 (9.1)	2 (15)	0 (0)	0 (0)
Missing	2 (6.1)						2 (22.2)
**Sex**, ***n*** **(%)**							
Male	26 (79)	9 (82)	2 (100)	16 (73)	9 (69)	7 (78)	8 (88.9)
Female	7 (21)	2 (18)	0 (0)	6 (27)	4 (31)	2 (22)	1 (11.1)
Presence crust, *n* (%)	8 (24.2)	NA	NA	NA	7 (53.9)	1 (11.1)	NA
Days,^†^ [mean (SD)]	2.9 (4.1)	NA	NA	NA	5.5 (5.7)	1.2 (1.2)	NA

† Average days between the onset of fever and rashes.

NA, not available.

In the published article, there was an error in the second line of the first paragraph of the results section.

A correction has been made to the second line of the first paragraph of the results. This sentence previously stated:

“Males accounted for 79% (28)…”

The corrected sentence appears below:

“Males accounted for 79% (26)…

The authors apologize for this error and state that this does not change the scientific conclusions of the article in any way. The original article has been updated.

