# Supplementary material for: The epidemiological trend of monkeypox and monkeypox-varicella zoster viruses co-infection in North-Eastern Nigeria
Source: Front Public Health. 2022 Dec 15;10:1066589. doi: 10.3389/fpubh.2022.1066589 (PMC9797713; doi:10.3389/fpubh.2022.1066589)
Supplement: Supplementary file 1 [file Data_Sheet_1.docx]

**Supplementary Material**


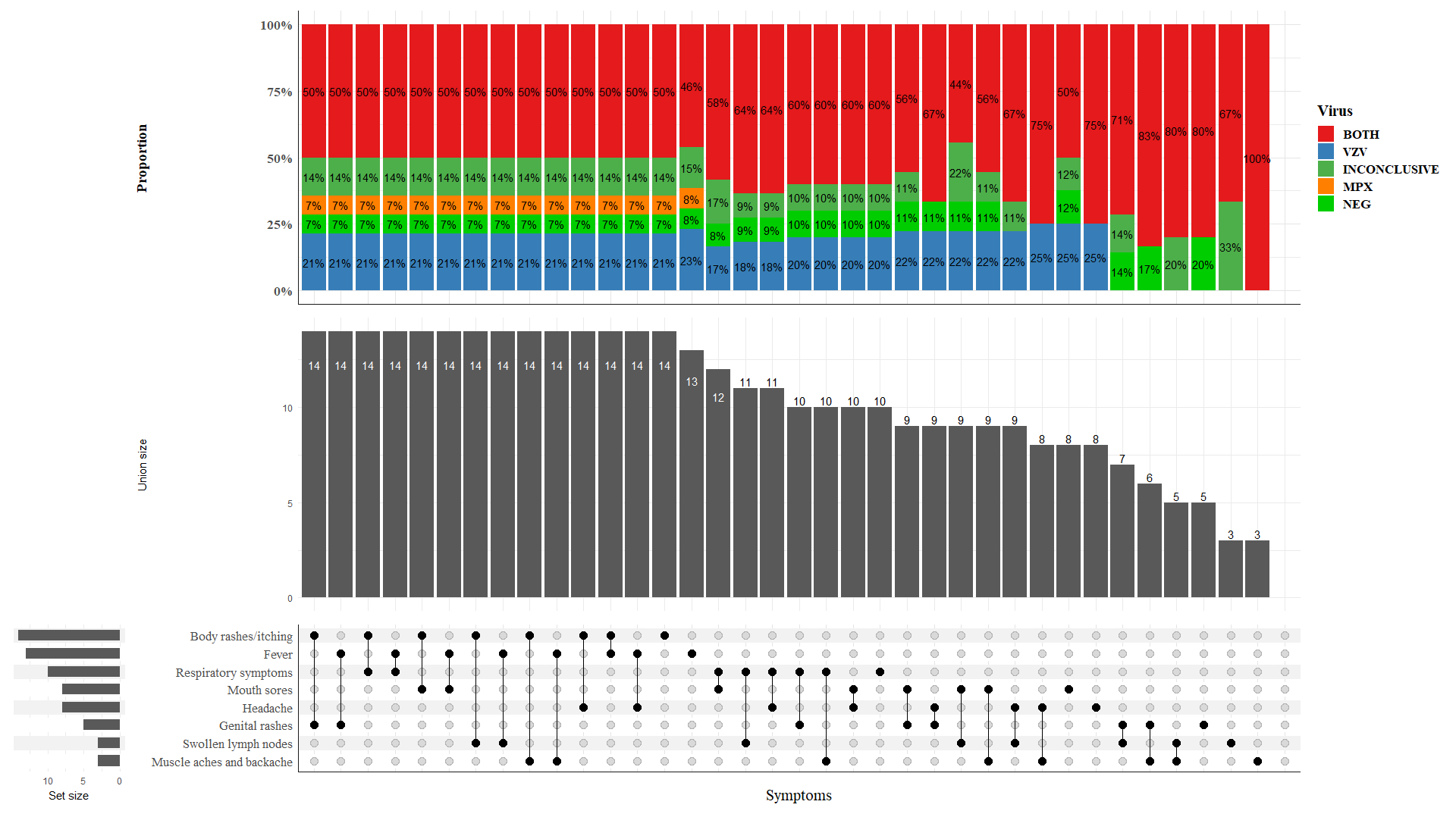


Figure S1. Clinical characteristics among 14 patients included in the follow-up survey for up to three symptoms at a time.


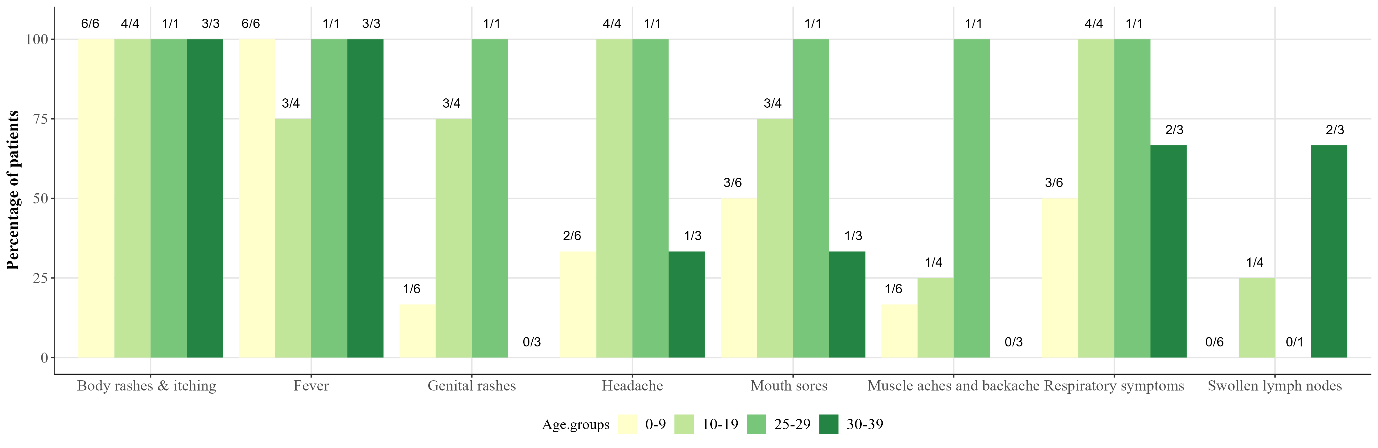


Figure S2. Bars represent the percentage of patients who experienced each symptom within their age group.
